# Environmental DNA adsorption to chitin can promote horizontal gene transfer by natural transformation

**DOI:** 10.1073/pnas.2420708122

**Published:** 2025-05-30

**Authors:** Jacob D. Holt, Yixuan Peng, Triana N. Dalia, Ankur B. Dalia, Carey D. Nadell

**Affiliations:** ^a^Department of Biological Sciences, Dartmouth, Hanover, NH 03755; ^b^Department of Microbiology and Immunology, Geisel School of Medicine at Dartmouth, Hanover, NH 03755; ^c^Department of Biology, Indiana University, Bloomington, IN 47405

**Keywords:** *Vibrio*, natural transformation, chitin, biofilm, pilus retraction force

## Abstract

Horizontal gene transfer by natural transformation (NT) is induced in *Vibrio cholerae* upon attachment to chitin surfaces in the aquatic environment. Here, we show that free environmental DNA adsorbs to chitin surfaces under physiologically realistic conditions. Using live-cell imaging and a fluorescent NT reporter, we demonstrate with cellular resolution microscopy that *V. cholerae* utilizes chitin-bound DNA as a reservoir for genetic exchange. Additionally, we demonstrate that uptake of DNA from this chitin-bound reservoir requires the forceful retraction of competence type IV pili. These findings uncover a role for retraction force in driving pilus-dependent NT and suggest that chitin particle surfaces can act as hotspots for horizontal gene transfer.

## Significance

The human pathogen and marine microbe *Vibrio cholerae* has been shown to activate extracellular DNA uptake and transformation when in contact with chitin, the most abundant structural biomaterial in the oceans and a primary source of nutrition for *V. cholerae* when living in aquatic habitats. Here, using realistic conditions, we show that environmental DNA adsorbs to chitin particles and that this DNA is then available to *V. cholerae* for uptake and recombination into the genome. We also demonstrate that the pilus retraction motor PilU, which previously had no known function in *V. cholerae*, is essential for retrieving chitin-bound DNA for natural transformation.

*Vibrio cholerae*, known for its pathogenicity in humans, is most commonly a marine microbe that forms biofilms on chitin particles, which it can consume as a source of carbon and nitrogen. Upon attachment to chitin, *V. cholerae* also upregulates genes for natural competence (1–3). Chitin-induced horizontal gene transfer by natural transformation (NT) plays an important role in the emergence of pathogenic serogroups (4, 5). While the genetic details underlying chitin-regulated NT are known, why *V. cholerae* activates NT upon attachment to chitin is less clear (6). Given that chitin is an insoluble biopolymer that can become positively charged when deacetylated, we hypothesized that it may facilitate horizontal gene transfer by binding DNA from the surrounding aquatic environment and thereby serve as a reservoir for genetic exchange by NT.

## Results and Discussion

### DNA Accumulates on Chitin under Flow.

To determine whether DNA adsorbs to chitin under flow, sterilized chitin flakes were loaded into microfluidic devices containing a region of post obstacles that trap the chitin in place. The chitin flakes were then exposed to a continuous volumetric flow rate of 0.1 µL/min (flow velocity of ~15 μm/s). The media used was defined artificial seawater, supplemented with either: Cy3-labeled DNA, Cy3-label alone, or unlabeled DNA alone. After 16 h of continuous flow, the chitin was imaged by confocal microscopy. The DNA concentration used here was 0.13 μg/L, which is conservative; for comparison, empirically measured DNA concentrations in the ocean range from 1.5 to 2.0 μg/L in shallow waters to 0.1 to 0.3 μg/L at greater depths (7).

**Fig. 1.**
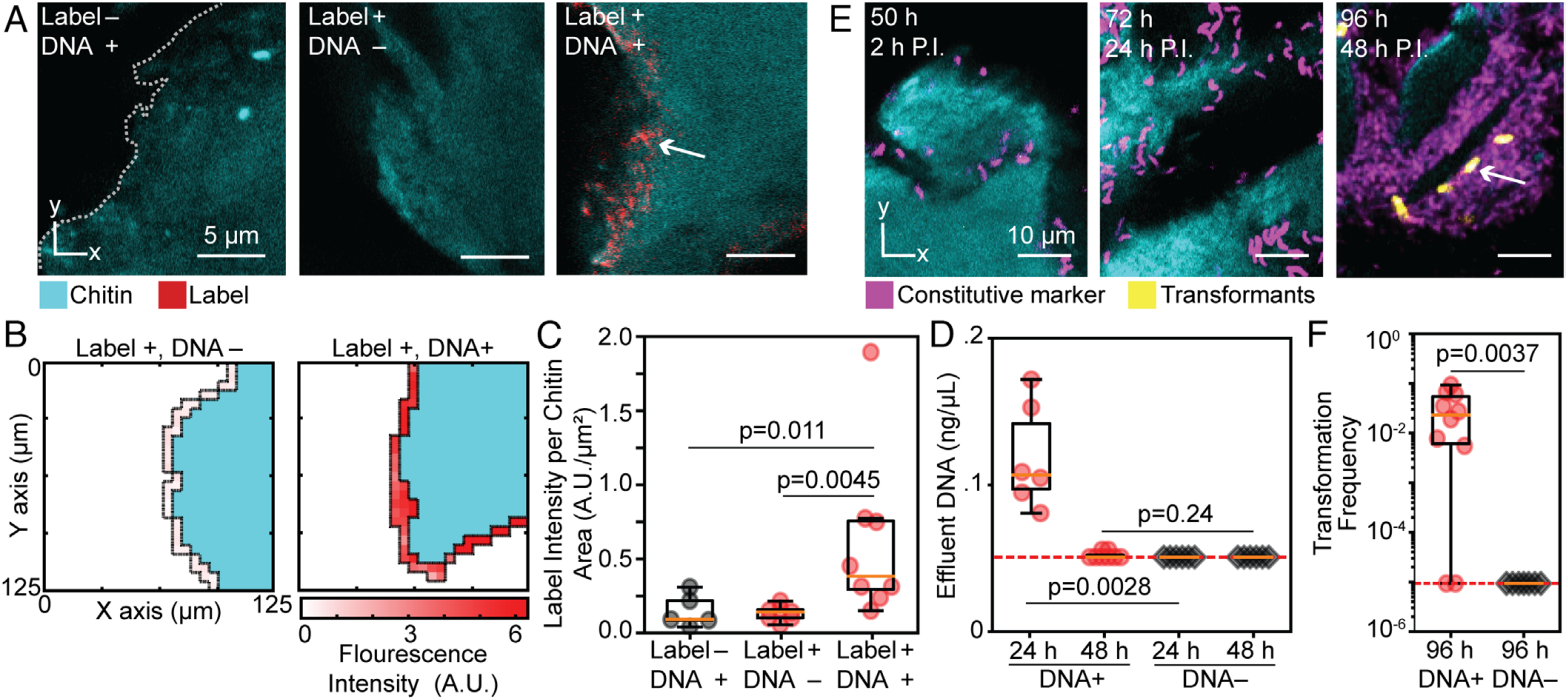
DNA accumulates on chitin under flow and is available for horizontal gene transfer by NT. (*A*) Representative images of chitin treated with DNA, Cy3 label, or Cy3-labeled DNA. At left, the outer surface of the chitin particle, which is exposed to the surrounding liquid, is shown with a dotted line trace. Note that the variable appearance of the chitin in panels *A* (and *E*), as well as in Fig. 2 and *SI Appendix*, Fig. S1, is due to confocal 2-dimensional optical sectioning through irregularly shaped 3-dimensional chitin particles used in the experiments. (*B*) Representative heatmaps quantifying Cy3 fluorescence intensity (A.U.) in proximity to the water-facing surface of sample chitin particles. (*C*) Box and whisker plots of Cy3 intensity, normalized to available chitin surface area (A.U./μm^2^), showing a significant difference between the Cy3-labeled DNA and Cy3 control (Mann–Whitney *U* test, n = 7-8) and the Cy3-labeled DNA and the unlabeled DNA control (Mann–Whitney *U* test, n = 5-8). (*D*) Box and whisker plot showing DNA concentration (ng/μL) in the effluent after a 24 h DNA addition period and a subsequent 24 h wash period. At 24 h, there is significantly more DNA in the effluent of the DNA treatment than in a control treatment to which no DNA was added (Mann–Whitney *U* test, n = 6). At 48 h, after the 24 h wash step, there is no significant difference in effluent DNA concentration between the two treatments (Mann–Whitney *U* test, n = 6-8). (*E*) Representative time series of images of *V. cholerae* biofilm formation and transformation on chitin. In these images, the time indicator at top left denotes the total elapsed time since the start of the experiment, and the time indicator just below denotes the elapsed time post inoculation with the *V. cholerae* reporter strain. Transformed cells first appeared 48 h post inoculation. (*F*) Box and whisker plot of *V. cholerae* transformation frequency 48 h after inoculation, indicating a significant increase in transformation frequency relative to a no-DNA control (Mann–Whitney *U* test, n = 7-10). Red dotted lines in panels (*D* and *F*) denote the limit of detection.

From the images, we immediately noted that the Cy3-labeled DNA treatment showed Cy3 signal along the water-facing surfaces of the chitin particles, while the controls displayed little to no signal on chitin particle surfaces (Fig. 1*A*). To quantify these observations, we summed the fluorescent signal of the label within 1 µm of the chitin particle outer surface, normalizing by the chitin surface area in each imaging sample. Representative heatmaps of the chitin signal and Cy3 label indicate an increase in fluorescence along the chitin outer surface in the Cy3-labeled DNA treatment relative to the control treatments (Fig. 1*B*). Across replicates, the Cy3-labeled DNA treatment displayed significantly higher fluorescent signal when compared the Cy3-only treatment or to the DNA-only treatment (Fig. 1*C*). To confirm that this result was not due to the preparation process for research-grade chitin, we recapitulated the experiment using particles of chitin shell derived with minimal processing from wild-caught, unfrozen shrimp (*SI Appendix*, Fig. S1). These results are consistent with the hypothesis that chitin adsorbs free DNA from the surrounding aqueous environment; having determined that DNA accumulates on chitin, we next sought to test whether chitin-adsorbed DNA is available to *V. cholerae* cells for NT.

### *V. cholerae* Utilizes Chitin-Adsorbed DNA for Horizontal Gene Transfer by NT.

To determine whether DNA adsorbed to chitin surfaces can be retrieved by *V. cholerae* for NT, we first confirmed that DNA could be removed from the bulk liquid media of the microfluidic device via a wash step. As described above, we allowed DNA to accumulate on chitin by flowing artificial seawater supplemented with 0.13 μg/L DNA through chitin-loaded microfluidic devices at 0.1 μL/min for 24 h. After this DNA accumulation phase, the chitin was washed with sterile artificial seawater for 24 h to remove any unbound DNA. To verify the wash, we used a Qubit fluorometer to measure the DNA concentration in the liquid exiting the chambers. At 24 h, immediately after the treatment with artificial seawater containing DNA, the effluent contained a significantly higher concentration of DNA than a no-DNA control. At 48 h, after 24 h of washing with artificial seawater, the DNA concentration in the bulk media dropped to the limit of detection (Fig. 1*D*), indicating that unbound DNA was removed from the chamber. Following the wash, we introduced a reporter strain of *V. cholerae* carrying a nonfunctional GFP construct and a functional, constitutive mKate2 construct. The DNA introduced into the chambers repairs the GFP reporter if integrated into the genome by NT (8). In this and following experiments, all cells are mKate2-positive, while cells that undergo NT (transformants) become GFP-positive as well. We first began to see GFP-expressing transformants that were closely associated with the chitin surface 48 h after inoculating the chambers with the reporter strain, establishing that chitin-adsorbed DNA is retrievable by *V. cholerae* for NT (Fig. 1 *E* and *F*). Though transformed cells occasionally fell off the chitin surface, most transformed cells were found in direct proximity to the chitin surface (*SI Appendix*, Fig. S2).

Chitin is commonly bound by other *Vibrios*, and prior work has demonstrated that *V. cholerae* can use type VI secretion and lytic activation of its temperate phages to kill neighboring *Vibrios* and utilize their genomic DNA as a substrate for NT (9, 10). Additionally, free DNA is found in oceanwater at the same (and higher) concentrations as those used here; based on our results, we speculate that most of the free chitin in the water column is coated with DNA sampled from various organisms in the surrounding environment (7). Our results establish that DNA adsorbed to chitin can be leveraged as a pool of genetic information for horizontal gene transfer within and potentially between species. Our results also offer a simple intuition for why *V. cholerae* evolved to upregulate competence machinery in response to chitin. Even if other *Vibrios* are not present, colonizing cells can still gain a potential benefit from upregulating competence, because there is likely to be chitin-adsorbed DNA from a variety of sources that can be sampled via NT for recombination—given sufficient sequence similarity to the native *V. cholerae* genome.

### The Force-Generating Retraction Motor, PilU, Is Required for Efficient NT When DNA Is Adsorbed to Chitin.

**Fig. 2.**
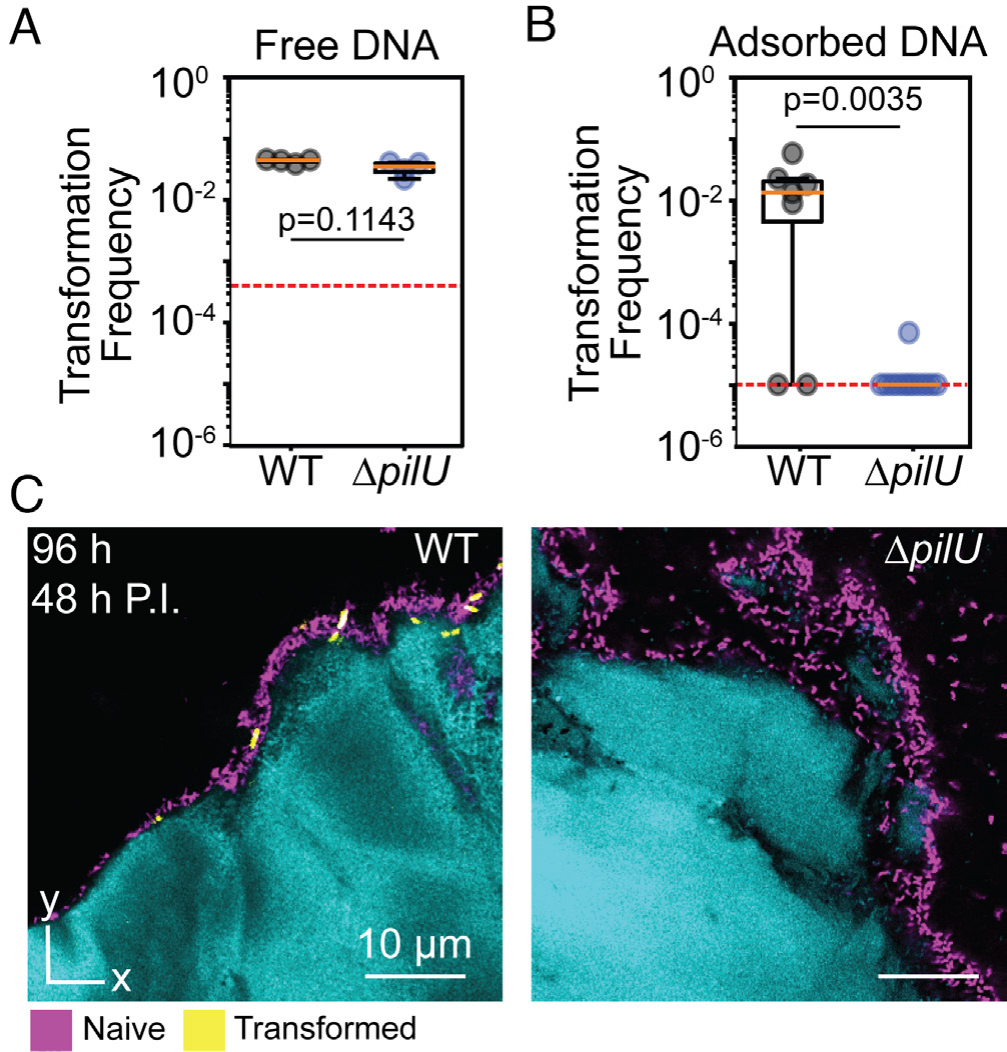
PilU is required for efficient NT when DNA is adsorbed to chitin. (*A*) A box and whisker plot showing no change in transformation frequency between WT and *ΔpilU* when assayed with DNA that is present in shaken liquid media (Mann–Whitney *U* test, n = 4). (*B*) A box and whisker plot illustrating the nearly complete loss of NT in a *ΔpilU* strain relative to WT when they are inoculated onto DNA-coated chitin particles (Mann–Whitney *U* test, n = 7-12). (*C*) Representative images of WT and *ΔpilU* biofilms. The red dotted lines in panels (*A* and *B*) denote the limit of detection.

*V. cholerae* utilizes dynamic surface appendages called competence type IV pili (T4P) to take up DNA from the environment during NT (11). These appendages bind DNA via their tip and then retract to pull this tip-bound DNA across the outer membrane (12). We and others have previously shown that the force-generating accessory ATPase motor, PilU, is dispensable when freely soluble DNA is provided as a substrate for NT in liquid culture (13, 14), a finding we recapitulate here (Free DNA; Fig. 2 *A* and *C*). We hypothesized that the biophysical requirements for DNA uptake may differ when DNA is adsorbed to chitin; specifically, we speculated that PilU-dependent high force retraction is required for NT when retrieving DNA that is adsorbed to chitin. Indeed, NT of a ∆*pilU* mutant is nearly eliminated when DNA is adsorbed to chitin, whereas in liquid culture with free DNA, the ∆*pilU* deletion has no measurable effect on transformation frequency (Fig. 2 *A*–*C*). Notably, there was no significant difference in WT transformation frequency when DNA was adsorbed to chitin particles vs. freely soluble (*SI Appendix*, Fig. S3), indicating that *V. cholerae* can efficiently sample chitin-adsorbed DNA. For other species such as *Pseudomonas aeruginosa*, PilU-dependent retraction has been shown to play an important role in T4P behaviors such as twitching motility (15). Our results uncover that PilU is necessary for efficient retrieval of surface-adsorbed DNA by competence T4P.

## Methods Summary

All bacterial strains were derived from *V. cholerae* E7946. Biofilm growth experiments were performed using polydimethylsiloxane microfluidic devices containing pillar obstacles to hold chitin particles in place for the duration of the experiments. Microscopy was performed using a Zeiss 880 point-scanning confocal microscope with a 40×/1.2 N.A. water immersion objective. Image data were quantified using MatLab and the BiofilmQ imagine processing framework. Further data analysis, statistical tests, and figure generation were performed in Python. A detailed methods description can be found in *SI Appendix*, *Materials and Methods*. The data used for the figures in the main text and SI are freely available for download (16).

## Supplementary Material

Appendix 01 (PDF)

## Data Availability

The study data have been deposited in a GitHub repository (https://github.com/nadellinsilico/DNA_adsorption_chitin_NT_2025) (16). All other data are included in the manuscript and/or *SI Appendix*.
