# Supplementary material for: Environmental DNA adsorption to chitin can promote horizontal gene transfer by natural transformation
Source: Proc Natl Acad Sci U S A. 2025 May 30;122(22):e2420708122. doi: 10.1073/pnas.2420708122 (PMC12146716; doi:10.1073/pnas.2420708122)
Supplement: Supplementary file 1 — Appendix 01 (PDF) [file pnas.2420708122.sapp.pdf]

Supplemental Information:

Environmental DNA adsorption to chitin can promote horizontal gene transfer by natural transformation

Jacob D. Holt[1,2], Yixuan Peng[1,2], Triana N. Dalia[3], Ankur B. Dalia[3*], Carey D. Nadell[1,2*]

[1] Department of Biological Sciences, Dartmouth, Hanover, New Hampshire, USA
[2] Department of Microbiology and Immunology, Geisel School of Medicine at Dartmouth, Hanover, New Hampshire, USA
[3] Department of Biology, Indiana University, Bloomington, Indiana, USA

[*]Authors for correspondence:

Carey D. Nadell
Class of 1978 Life Science Center
78 College St.
Hanover, NH 03755
USA.
Email: carey.d.nadell@dartmouth.edu

Ankur B. Dalia
Indiana University
Biology Building
1001 E Third St.
Bloomington, IN 47405
USA.
Email: ankdalia@iu.edu

Author Contributions: ABD and CDN conceived, funded, and supervised the study. JDH, ABD, and CDN designed experiments. JDH, YP, and ABD performed experiments, and JDH generated the figures. TND and ABD provided key reagents and strains. JDH, ABD, and CDN wrote the paper.

Competing Interest Statement: The authors have no competing interests to declare

Classification: Microbiology

Keywords: *Vibrio*, Natural transformation, Chitin, Biofilm, Type 4 pilus, Pilus retraction force

This file includes:

- SI Figures
- List of strains, materials, and software
- SI Methods
- SI references

SI Appendix, Materials and Methods

List of strains, materials, and software.

**Bacterial strains**	**Relevant markers/Genotype**	**Source**
CNV268 / TND3433	E7946 Sm^R, ΔVC1807::Cm^R-P_{tac}-*gfp*::parS$_{MT1}$*, Δ*lacZ*::$Spec^R$-P_{tac}-*mKate2*	This study
CNV269 / TND1166	E7946 SmR, ΔVC1807::Cm^R-P_{tac}-*gfp*, Δ*luxO*, Δ*lacZ*::*lacIq*, *pilA^{S67C}*, P_{tac}-*tfoX*	(1)
CNV310 / TND4366	E7946, Δ*pilU*::Tm^R, ΔVC1807::Cm^R-P_{tac}-*gfp*::parS$_{MT1}$*, Δ*lacZ*::$Spec^R$-P_{tac}-*mKate2*	This study
**DNA oligos**	**Sequence**	**Description**
CNO837	GTATCGATCAGCAAGGACAAC	Amplify "ΔVC1807::Cm^R-P_{tac}-*gfp*" to generate tDNA
CNO838	CAATTTTGCTTTTGGACCATCCC	Amplify "ΔVC1807::Cm^R-P_{tac}-*gfp*" to generate tDNA
**Chemicals and reagents**	**Source**	**Product Number**
Chitin from shrimp shells	Thermo Scientific Chemicals	cat. #C7170
Shrimp	Gulf of Mexico	N/A
Instant Ocean	Marineland	cat. #AQ-SS350 /11-23919
NaCl	Sigma-Aldrich	cat. #S271
$MgSO_4$	Sigma-Aldrich	cat. #M2643
$CaCl_2$	Sigma-Aldrich	cat. #C8106
KCl	Sigma-Aldrich	cat. #P9333
$NaHCO_3$	Sigma-Aldrich	cat. #S5761
$Na_2B_4O_7$	Sigma-Aldrich	cat. #B9876

SrCl	Sigma-Aldrich	cat. #255521
NaI	Sigma-Aldrich	cat. #746371
LiCl	Sigma-Aldrich	cat. #L4408
NaBr	Sigma-Aldrich	cat. #S4547
K_2HPO_4	Sigma-Aldrich	cat. #5504
Triethanolamine	Sigma-Aldrich	cat. #T9534
IPTG	Sigma-Aldrich	cat. #I67578
Labeling IT Nucleic Acid Labeling Kit, Cy3	Mirus Bio	cat. #MIR3625
Qubit dsDNA Quantification Kit, High Sensitivity	Thermo Fisher	cat. #Q32854
Platinum™ SuperFi II Green PCR Master Mix	Thermo Fisher	cat. #12369010
PCR Purification Kit	QIAquick	cat. #28104
Poly-dimethysiloxane (PDMS)	Dow Chemical Company SYLGARD 184	cat. #04019862
#1.5 glass coverslips	Azer Scientific	cat. #1152260
Inlet tubing	Cole Palmer	cat. #06417-11
27Gx1/2 needles	BD Precision	cat. #30510
1 mL syringes	Brandzig	cat. #CMD2583
Harvard Apparatus Pico Plus Elite syringe pumps	Harvard Apparatus	cat. #70-4506
**Software and algorithms**	**Source**	**Version**
ZEN Black	Zeiss	Version 2.3
ZEN Blue	Zeiss	Version 2.3
Nikon NIS Elements	Nikon	Version 5.00
MATLAB	MathWorks (2)	Version R2021a
BiofilmQ	drescherlab.org (3)	Version 0.2.2
Python	Python.org (4)	Version 3.8.8
Anaconda	Anaconda.org	Version 2021.05
Spyder	Spyder-ide.org	Version 4.2.5

Pandas	Pandas.pydata.org (5)	Version 1.6.2
NumPy	NumPy.org (6)	Version 1.20.1
Matplotlib	Matplotlib.org (7)	Version 3.3.4
SciPy	Scipy.org (8)	Version 1.6.2
ImageJ	Imagej.net (9)	Version 2.0.0
MicrobeJ	Microbej.com (10)	Version 5.13

Methods

Bacterial strains

The *V. cholerae* strains used in this study were derived from E7946 (11). The fluorescent protein expression constructs and *ΔpilU* mutant were made here and previously using standard methods. Cultures were grown overnight in lysogeny broth (LB) with the addition of 100 µM IPTG. Microfluidic flow cultures were grown in defined artificial seawater media (DASW) with the addition of 100 µM IPTG and chitin flakes from shrimp shells; for bulk shaken liquid culture work, we used instant ocean (IO). Prior to injecting into microfluidic devices via pipette, chitin was washed with 70% EtOH and defined artificial seawater (DASW). Defined artificial seawater consists of 234 mM NaCl, 27.5 mM $MgSO_4$, 1.5 mM $NaHCO_3$, 4.95 mM $CaCl_2$, 5.15 mM KCl, 0.07 mM $Na_2B_4O_7$, 0.05 mM SrCl, 0.015 mM NaBr, 0.001 mM NaI, 0.013 mM LiCl, 0.187 mM K_2HPO_4, and pH 7.1 triethanolamine.

Microfluidic device assembly

Microfluidic devices were made with polydimethylsiloxane (PDMS) using standard soft lithography techniques. PDMS was mixed with its curing agent at a ratio of 10:1 (PDMS/curing agent); this mixture was then de-gassed using a vacuum chamber and poured into chamber molds to solidify. After cutting the chambers to size for a coverslip, holes were punched for inlet and outlet tubing, and the chambers were bound to #1.5 24 mm by 60 mm glass coverslips (Azer Scientific) via plasma cleaning. Continuous flow was generated via Harvard Apparatus Pico Plus syringe pumps loaded with 1 mL Brandzig plastic syringes. Syringes were affixed with 27-gauge needles that were fitted with #30 Cole Parmer PTFE tubing. The inlet tubing for each chamber within the microfluidic device was connected to a pump-mounted media syringe, and the outlet tubing was run into a petri plate for waste collection.

Fluorescence microscopy

Fluorescence imaging of flow-cell experiments was performed using a Zeiss 880 line-scanning confocal microscope and a 40x/1.2 N.A. water objective. The GFP protein expressed after homologous recombination of the exogenously introduced DNA was excited with a 488-laser line. The mKate2 protein was excited with a 594-laser line. All fluorescent protein constructs required IPTG for expression, so IPTG was continually supplied in the media for the pertinent experiments. The chitin flakes, which are auto fluorescent, were excited with a 405-laser line. The Cy3 DNA label was excited with a 543-laser line. Representative images containing bacterial cells were processed by constrained iterative deconvolution in ZEN blue. Fluorescence imaging of well-mixed experiments was performed using a Nikon Ti-2 microscope using a Plan Apo 60x objective, a YFP and/or mCherry filter cube, and a Hamamatsu ORCA Flash 4.0 camera, all controlled by the NIS Elements software.

Image analysis

For image stack data collected from flow-cells, Zeiss CZI files were converted to .tiff stacks and loaded into BiofilmQ v0.2.2, which was run using MATLAB v2021a. Biovolume thresholding of bacterial cells and chitin was performed using the Robust Background algorithm with a manual sensitivity adjustment. The quantification of Cy3 label was done by segmenting the chitin flake autofluorescence signal and then summing the fluorescence intensity of Cy3 signal (A.U.) within 1 µm of the chitin surface. Transformation frequency was calculated by dividing the biovolume of GFP expressing cells (transformed cells) by the

biovolume of mKate2 expressing cells (total bacterial population). For all experiments, biological replicates were defined as independent chambers, while technical replicates include independent images from within the same chamber. For data collected from well-mixed culture tubes, images were analyzed using the MicrobeJ plugin in FIJI to determine the number of GFP-positive cells (transformants) and mKate2-positive cells (total cells) to define the transformation frequency. At least 2500 cells were analyzed per replicate.

Replication and statistics
The Mann-Whitney *U* test was used for all pairwise comparisons. Biological replicates were independent microfluidic device channels, each with 1-3 chitin flakes averaged as technical replicates within each chamber at each time point. All box and whisker plots were generated in Python v3.8.8 using Matplotlib v3.3.4. All statistical test comparisons were performed in Python using SciPy v.1.6.2. Python was run in Spyder v4.2.5 using Anaconda v2021.05. Numpy v1.20.1 and Pandas v1.6.2 were used for datasheet file handling. The raw data and code used to generate graphs and statistics are available on GitHub.

Labeled DNA assay
Chitin flakes were incubated with ethanol for 1 min prior to being washed 5x with PBS and 2x with DASW. The chitin flakes were then loaded into microfluidic devices containing a region of pores that trapped the chitin flakes. After loading the chitin, the treatment media was applied to the chitin for 16 h at a volumetric flow rate of 0.1 µL/min (corresponding to an average flow velocity of 15 µm/s). The treatment media consisted of DASW containing 0.13 ng/µL of Cy3-Labeled 9.9 kb DNA (~200 femtomoles of DNA bp and ~5 femtomoles of Cy3 labels per µL), DASW containing ≥ ~5 femtomoles of Cy3 labels per µL, or DASW containing 9.9 kb DNA (~200 femtomoles of DNA bp per µL). After 16 h of exposure to one of the three media conditions, the chambers were imaged. The DNA was labeled using the Mirus DNA Labeling Kit. In brief, the buffer, Cy3-Label, DNA, and water were incubated before the labeled DNA was purified using a G50 spin column. For the Cy3 control, the DNA labeling protocol was replicated but without the addition of DNA or the purification step. This resulted in as much, if not more, Cy3 label being present in the Cy3 label control group as in the Cy3 labeled DNA group. Images were taken at least 3 µm above the glass coverslip surface to avoid collecting signal from the glass. The chitin flakes used in main text experiments were purchased from Thero Scientific Chemicals. The chitin shell used in SI Figure S1 was derived from wild caught shrimp from the Gulf of Mexico, purchased from the Hanover, NH Co-op Food Store. To process chitin for imaging, shrimp shells were peeled before being placed in PBS and bead-bashed using a mixture of silicone and chrome beads for 1 minute.

Flow-cell natural transformation assay
The chitin flakes were loaded into the microfluidic chambers as described above and then subjected to a 24 h wash at a volumetric flow rate of 0.1 µL/min, with either DASW containing 0.13 ng/µL of DNA or a DASW control containing no DNA. After 24 h, the inlet tube was replaced with media containing only DASW to wash any un-bound DNA out of the flow device. The DNA present in the effluent was quantified at times 24 h and 48 h (immediately post DNA+DASW wash and immediately post plain DASW wash). All DNA quantification was done using a Qubit 2.0 with the high sensitivity double-stranded DNA kit. In brief, 12 µL of effluent was collected for quantification over the course of 120 min. 2 µL of effluent was then diluted into the Qubit master mix (buffer and fluorescent label) and quantified. At time 48 h, after 24 h of wash with blank DASW, 100 µL of a strain carrying a mKate2 protein and the 'broken' GFP NT reporter was added to the chitin flakes at an OD_{600} of 5.0. Live-cell imaging was then performed at times 2 h, 24 h, and 48 h post inoculation (see figure captions). All flow-cell experiments were performed at room temperature (~22°C).

Well-mixed natural transformation assay
V. cholerae strains were grown overnight in LB with shaking at 30°C. Then, 13 µL of the overnight culture was subcultured into 3 mL of LB and grown with shaking at 30°C to an OD_{600} of ~1.0. Cells were then spun down and washed in 0.5x instant ocean (IO) and resuspended to an OD_{600} of 1. Then, 100 µL of cells were mixed with 150 µL of chitin slurry (8 g of chitin flakes autoclaved in 150 mL 0.5x IO) and 750 µL of 0.5x IO for each reaction. Reactions were then incubated static overnight at 30°C to allow the cells to begin digesting the chitin and become competent. The following day, 550 µL of media was removed without disturbing the settled chitin, which contained the competent cells. Reactions were then heavily vortexed for 1 min to lift cells off the chitin flakes. Then, 1000 ng of transforming DNA was added to reactions. Reactions were then incubated at 30°C overnight with shaking at 250 rpm to ensure that cells and DNA remained in

suspension. The following day, reactions were once again vortexed for 1 min and then left to sit for 2 min to allow the chitin to settle. Then, 150 µL of the supernatant was transferred to a fresh tube. This mixture was spun at 18,000 x g for 1.5 min to pellet the cells. The cells were then resuspended in 10 µL of 0.5x IO and placed under a gelzan pad for imaging.

Supplementary Figures

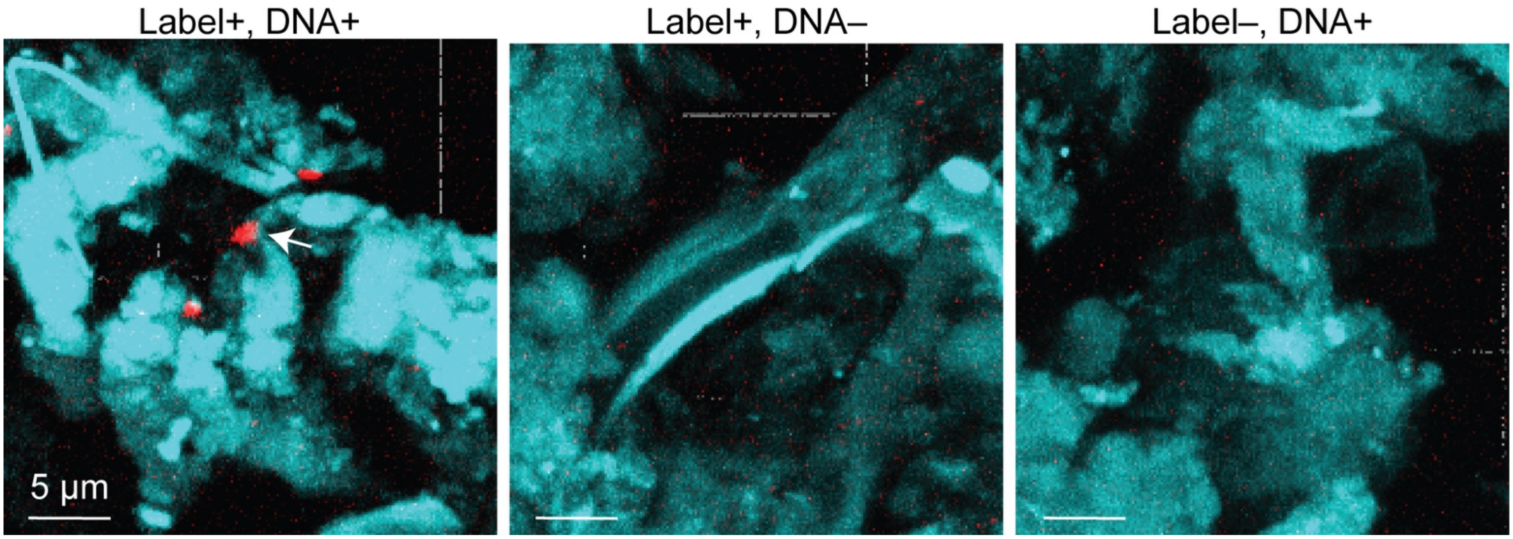

Figure S1. Labeled DNA adsorbs to chitin shell particles (shown in blue), which were minimally processed from wild-caught, unfrozen shrimp; this result captures the same qualitative trend observed in Figure 1A. The left-hand panel illustrates labeled DNA localization to the chitin surface (red puncta); the middle and right-hand panels are label-only and DNA-only controls, respectively. Note that the variation in the shape of the chitin.

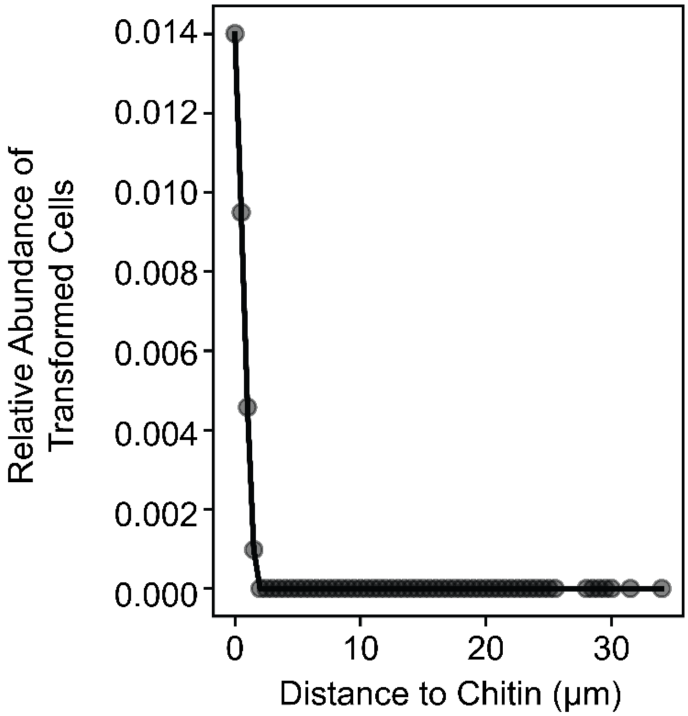

Figure S2. A frequency distribution plot of transformed cells' location as a function of distance from the outer surface of chitin particles. This quantification demonstrates that transformed cells are predominantly located directly on or adjacent to the chitin surface.

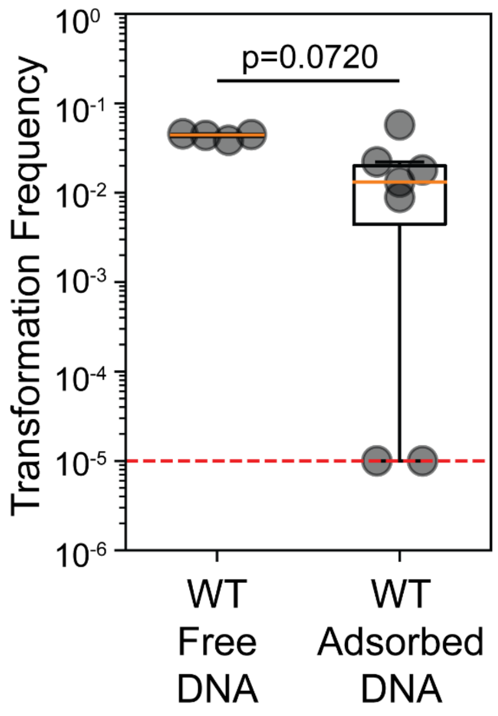

Figure S3. A side-by-side comparison of natural transformation frequency for wildtype *V. cholerae* (data recapitulated from Fig. 2A-B) when DNA is scavenged from the surface of chitin particles, versus shaken liquid culture conditions in which both cells and DNA are freely intermixed in the liquid phase. The difference in natural transformation frequency between these two conditions is not statistically significant. (Mann-Whitney U-test with n=4-7, data recapitulated from two panels in Figure 2 for ease of comparison).

References

1. A. B. Dalia, T. N. Dalia, Spatiotemporal Analysis of DNA Integration during Natural Transformation Reveals a Mode of Nongenetic Inheritance in Bacteria. *Cell* **179**, 1499-1511.e10 (2019).

2. T. M. Inc, MATLAB version: 9.13.0 (R2021a). (2022). Deposited 2022.

3. R. Hartmann, *et al.*, Quantitative image analysis of microbial communities with BiofilmQ. *Nat Microbiol* **6**, 151–156 (2021).

4. M. Pilgrim, S. Willison, *Dive Into Python 3* (Springer, 2009).

5. T. pandas development team, pandas-dev/pandas: Pandas. (2020). https://doi.org/10.5281/zenodo.3509134. Deposited February 2020.

6. S. van der Walt, S. C. Colbert, G. Varoquaux, The NumPy Array: A Structure for Efficient Numerical Computation. *Comput. Sci. Eng.* **13**, 22–30 (2011).

7. J. D. Hunter, Matplotlib: A 2D Graphics Environment. *Comput. Sci. Eng.* **9**, 90–95 (2007).

8. SciPy 1.0 Contributors, *et al.*, SciPy 1.0: fundamental algorithms for scientific computing in Python. *Nat Methods* **17**, 261–272 (2020).

9. C. A. Schneider, W. S. Rasband, K. W. Eliceiri, NIH Image to ImageJ: 25 years of image analysis. *Nat Methods* **9**, 671–675 (2012).

10. A. Ducret, E. M. Quardokus, Y. V. Brun, MicrobeJ, a tool for high throughput bacterial cell detection and quantitative analysis. *Nat Microbiol* **1**, 16077 (2016).

11. M. M. Levine, *et al.*, The Pathogenicity of Nonenterotoxigenic Vibrio cholerae Serogroup 01 Biotype El Tor Isolated from Sewage Water in Brazil. *Journal of Infectious Diseases* **145**, 296–299 (1982).
